# *Alternations (at) that time*: NP versus PP time adjuncts in the history of English

**DOI:** 10.1515/lingvan-2023-0054

**Published:** 2024-01-09

**Authors:** Eva Zehentner

**Affiliations:** Department of English, University of Zurich, Zurich, Switzerland

**Keywords:** NP versus PP, prepositions, adjuncts of time, history of English

## Abstract

The present paper investigates variation between nominal and prepositional adjuncts of time as in, for example, [*on*] *that day, they left*. The main goals are (i) to assess potential changes in the distribution of these variants in the history of English, specifically from Middle English to Late Modern English (1150–1914), and (ii) to test which factors most strongly impact the choice between the two variants, with a focus on the impact of different complexity measures. To address these questions, the paper makes use of data from the Penn-Helsinki Parsed Corpora of Historical English (PPCME2; PPCEME; PPCMBE), explored by means of logistic regression modelling. The results suggest that there is no dramatic, sweeping change in this abstract alternation over time, but that this variation may mainly plays out on lower, noun-specific levels.

## 1 Introduction

This paper contributes to the study of alternations in diachrony, focusing on two central questions in any such investigation. These are (i) whether any changes can be detected in the history of an alternation, including the emergence or loss of an alternation, and (ii) which factors impact the choice between variants over time (cf. e.g. Wolk et al. [2013] for a comparable investigation into the dative and genitive alternation). Following Pijpops et al. (2021), the paper also addresses the issue of determining the appropriate locus of an alternation, viz. the question on which level of schematicity or abstractness an alternation plays out.

The specific case study the paper homes in on is the expression of adjuncts of time and their development in the history of English since the Middle English period. As can be seen in the examples below, in Present-Day English references to points in time, as well as appearing in other structures such as adverbial phrases, can be made by means of an NP, as in (1), or a PP involving different prepositions, such as *on* in (2a) or *at* in (2b); according to Huddleston and Pullum (2002: 696), using PPs is the most common option. This is mirrored in Quirk et al. (1985: 489–501), who find that PPs account for about two-fifths of all adverbials (including, but not restricted to temporal adjuncts) in the Survey of English Usage Corpus, while the proportion of NP expressions is exceedingly smaller in comparison.

(1)

**Table:** 

a.	*She had made no prior purchases **that day***. (Corpus of Contemporary American English, COCA, 2012; ebcitizen.com)

(2)

**Table:** 

a.	*There was an enormous amount of emotion **on that day*** (COCA, 2012; newyork.cbslocal.com)
b.	*That’s pretty frightening. What did you see* ***at that day****?* (COCA, 1993; *Amtyville: A New Generation*)

Despite their differences in overall frequency, the two variants seem to exhibit similar positional preferences. Both may appear in either clause-initial (pre-verbal) or clause-late (post-verbal) position, as also visible in the comparison between (1)–(2) and (3); however, final position is found in a majority of cases with NPs and with PPs (Quirk et al. 1985: 500–501; also Ernst 2002; Hasselgård 2010). Medial position is rare but not impossible for both NP and PP adjuncts (De Clerq et al. 2012; Haegeman 1983, 2002).

(3)

**Table:** 

a.	***That day** I saw them for three hours* (COCA, 2012; tinyarticle.com)
b.	***On that day****, I did not go with them* (COCA, 2012; Demand EUPHORIA)

As for semantics, the patterns in question “normally locate in time the situation expressed by the verb together with its complements” (Huddleston and Pullum 2002: 694). This may be done deictically (relating to the time when a sentence is uttered, as in *last week*), but may also be relative to other time frames (e.g. *on the same day*), or refer to specific points of orientation such as calendar dates or clock times (*on the 1st of August*; see e.g. Huddleston and Pullum 2002: 695–696).

Most importantly for the present paper, this case of variation has arguably undergone change over time in the history of English. While nominal time adjuncts presumably constitute the original, earlier variant, and are frequent in the earliest English documents (see e.g. Sato 2009), PPs appear to have only gradually expanded in their functions, increasingly coming to compete with NP patterns over time (Traugott 1992: 207). This development forms part of a presumed general increase in more analytic patterns at the expense of more synthetic structures since Old English, viz. a loss of morphological inflections concomitant with a rise in prepositional means of expression, among other things (Baugh and Cable 1993: 60; also e.g. Hawkins 2012). Examples (4a) and (4b) from Old English texts illustrate both types, with sentence (4b) moreover reflecting the greater positional flexibility of adjuncts at that time – as shown in a range of studies on constituent order in historical English such as Haeberli (2000, 2002, 2017, Bech (2001), and van Kemenade and Los (2006), among many others, medial adjuncts were still comparatively frequent in earlier periods, but have decreased over time as a consequence or reflection of growing restrictions in ordering.

(4)

a.

**Table:** 

***þæs geares** þe Crist acenned wæs*
‘the year that Christ was born’
(ÆCHomi.80.30; Mitchell 1985: 586)

b.

**Table:** 

*Com **on wanre niht** scriðan sceadugenga*
‘There came in a gloomy night striding the shadowgoer’
(Beo.Th.1410; B.703; Bosworth 2014, s.v. *scríðan*)

Unlike these earlier diachronic studies, which focus particularly on constituent order and are often restricted to one variant only, the present paper approaches adjuncts (and specifically adjuncts of time) with an emphasis on the alternation relationship between NPs and PPs, and potential changes in both the distribution of the variants as well as the factors guiding the alternation over time. The key hypotheses to be tested are (i) that prepositional adjuncts of time increase from Middle English onwards, while NP constructions decrease in comparison, and (ii) that both patterns show differences in their syntactic and semantic features, which may also be subject to change.

The investigated factors include lexical biases (with different head nouns exhibiting distinct preferences), the position of the time NP/PP relative to the verb, as well as several “complexity” factors: morphosyntactic complexity, constituent length, distance between predicate and time NP/PP, and collocational strength. This follows Levshina’s (2018) investigation of complexity and efficiency effects in the variation between nominal and prepositional patterns with *home*, which shows that Rohdenburg’s (1996) Complexity Principle applies to this particular case of variation – more explicit formal structures (the prepositional variant) are preferred in cognitively more complex contexts; for example, in instances where the predicate is further removed from the constituent in question. By contrast, when the two elements are close in position, the NP variant is more frequent (Levshina 2018: 83). Furthermore, predictability or information content (operationalized as the collocational strength between a given verb and *(at) home*) influences the choice, as more “promiscuous” verbs are found more often in the PP construction, while verbs more strongly associated with the phrase favour the nominal option (Levshina 2018: 83, 85–86). That is, verbs frequently occurring with this lexical item (among all uses of the respective verbs), such as *stay*, are taken to be more predictable in this context; verbs used infrequently with *home* are less identifiable. The study’s results then indicate that the shorter or less explicit NP variant (*home*) “is preferred when either the verb or the adjunct is more predictable” (Levshina 2018: 72), whereas lower predictability instances constitute a more complex environment and accordingly favour the more explicit PP expression as a processing aid. Pijpops et al. (2018) evidence complexity effects in Dutch argument structure variation, finding that greater length of objects in pre-verbal position increases the probability of PP expressions – PPs are here also shown to reduce processing complexity in terms of individual prepositions limiting the number of possible verbs likely to follow.

Similar effects are expected to hold for the present case: grammatically more complex, longer, and more distant constituents are anticipated to increase the odds of the PP variant to be used, while the NP should appear in less complex, shorter, and closer contexts; moreover, some interaction with order or position is expected. As for potential change over time, the effects of all complexity measures are presumed to be constant; however, ordering tendencies and lexical preferences may vary. The hypotheses are tested by means of data extracted from corpora of Middle English, Early Modern English, and Late Modern (British) English, thereby covering a timespan from 1150 to 1914. In regard to methods, the paper uses mixed-effects logistic regression modelling (e.g. Winter 2019) as well as conditional random forests (e.g. Strobl et al. 2007, 2008).

The paper is structured as follows: Section 2 provides details on the data set used and the methods employed to analyse it. This is followed by the main results in Section 3, starting by reporting on the frequency distribution of the patterns in question over time (Section 3.1), before focusing on factors impacting the choice between variants (Section 3.2). Section 4 discusses the implications of the results for the questions posed, and concludes the paper.

## 2 Data and methods

The data for the present study is derived from a larger project on prepositions in argument structure from Middle English to Late Modern English (Zehentner et al. 2023), which comprises all instances of verbs (excluding modals, as well as *be*, *do*, and *have*) in a “sister-relation” to NPs and PPs in the Penn-Helsinki Parsed Corpus of Middle English (PPCME2; Kroch and Taylor 2000), the Penn-Helsinki Parsed Corpus of Early Modern English (PPCEME; Kroch et al. 2004), and the Penn-Helsinki Parsed Corpus of Late Modern British English (PPCMBE2; Kroch et al. 2016). These corpora have respective sizes of about 1.2, 1.8, and 2.8 million words, and include texts produced in 1150–1500, 1500–1710, and 1700–1914, respectively.

In the Penn-Helsinki Corpora annotation, NP arguments functioning as adjuncts of time are tagged as “NP-TMP”; however, a comparable tagging is not available for PPs. Therefore, the following steps were taken to retrieve relevant data for time adjuncts that are variable between both patterns. First, all temporal NPs were extracted from the data and a list of all head nouns in these NPs was compiled; then, the data set of all PP instances was compared to this list, and both sets were restricted to only those lemmas that were attested at least once in both NP and PP patterns across all periods. In order to ensure interchangeability (at least to some extent), the PP data set was next filtered to retain the prepositions *at*, *in*, *on*, and *upon*, but not others like *with*, *from*, or *to*, since these can presumably not alternate with an NP. Last, the list of head nouns was narrowed down further by excluding potentially polysemous or ambiguous elements such as *period* or *term*, all lexical items that were attested with a total frequency of less than 50 in the entire data set, and all those that were not attested in all periods (i.e. at least once in Middle English, Early Modern English, and Late Modern English).

This resulted in 14 remaining time nouns, given in Table 1 in alphabetical order together with the total number of each in the data set. The total number of instances included in the analysis is *N* = 9,043, of which 4,547 are NPs, and 4,496 are PPs. Note that the constituents may be “bare”, but may also show modification of any sort, as illustrated in examples (5a) and (5b) of an NP and a PP pattern from Middle English.

**Table 1:** Time nouns analysed in the study, together with their number of occurrences in the data set.

Time noun	Number of tokens
*Afternoon*	199
*Day*	2,408
*Hour*	174
*Month*	150
*Morning*	730
*Morrow*	101
*Night*	811
*Noon*	68
*Season*	108
*Summer*	75
*Time*	2,932
*Week*	153
*Winter*	75
*Year*	1,059

(5)

a.

**Table:** 

*the vj day aftyr that […] the iij heddys were takyn downe of london brygge*
‘the 6th day after that, the 3 heads were taken down off London Bridge’
(ME4, 1475; CMGREGOR,194.1502)

b.

**Table:** 

*o þatt illke herodess daȝ comm jesu crist to manne*
‘on that same Herod’s day came Jesus Christ to man’
(ME1, 1200; CMORM,I,7.183)

The data was then coded for a range of variables. These include, first, meta-information such as text ID, decade of manuscript production, and period (ME, EME, or LME), as well as a measure of “time” based on decade (centred around 1650 as the mean decade, log-transformed and scaled by means of 2 standard deviations; cf. Röthlisberger 2018: 87; also Gelman 2008). For each instance, the lemmas of the lexical items involved (verb, preposition, and head noun) are also specified. Furthermore, the factors of position and complexity were annotated in the following way:

(i) Position of NP/PP in relation to verb: rather than coding for absolute position in the clause, the time adjuncts were classified for either “post-verbal” or “pre-verbal” position.
(ii) Morphosyntactic complexity: a distinction is made between “simple” and “complex”, with the latter referring to NPs/PPs including a modifying relative clause or further complementation (e.g. by infinitives or gerunds and the like).
(iii) Length: this is the log-transformed, centred, and scaled length of the NP/PP constituent in words, excluding the preposition in the case of the latter.
(iv) Distance between verb and NP/PP: regardless of order of the elements, this measure gives the (log-transformed, centred, and scaled length) distance in words.
(v) Collocation strength: this provides the extent of attraction between individual verbs and head nouns, determined by means of multiple distinctive collexeme analysis and using t-score as the association measure (Stefanowitsch and Flach 2020; Stefanowitsch and Gries 2003).

These last four variables, viz. the complexity measures employed in this paper, are based (loosely) on Levshina (2018) and Pijpops et al. (2018), among others.

I used R (R Development Core Team 2021) to perform all analyses and visualization, specifically functions from the packages ggplot2 (Wickham 2016), dplyr (Wickham et al. 2018), and viridis (Garnier et al. 2021). Furthermore, the package collostructions (Flach 2021) was employed for measuring association strength as outlined in (v). The packages lme4 (Bates et al. 2015), car (Fox and Weisberg 2019), JGmermod (Grafmiller 2019), and effects (Fox and Weisberg 2019) as well as jasongraf1/JGmermod (Grafmiller 2019), were used for mixed-effects logistic regression modelling (Gelman and Hill 2007; Winter 2019: 232–273); the conditional random forest analysis was carried out by means of the package party (Hothorn et al. 2006), largely following Röthlisberger (2020).

The following sections first present the general distributional patterns in the data, addressing the question whether prepositional adjuncts of time increase at the expense of NPs from Middle English onwards. Afterwards, findings on the factors determining the choice between the patterns (and potential changes in their impact) are reported on.

## 3 Results

### 3.1 Frequency distribution over time

As can be seen in Table 2, the distribution of NP versus PP adjuncts is relatively balanced across periods, with a slight increase towards the latest period: in Middle English, both patterns account for roughly 50 % of the instances, whereas in Late Modern English, prepositional patterns are marginally more frequent at about 55 %.

**Table 2:** Absolute and proportional frequencies of NP and PP adjuncts across main periods.

Adjunct type	Middle English		Early Modern English		Late Modern English	
	*N*	%	*N*	%	*N*	%
NP	941	51.14	1,732	56.31	1,874	45.41
PP	899	48.86	1,344	43.69	2,253	54.59

**Figure 1:**
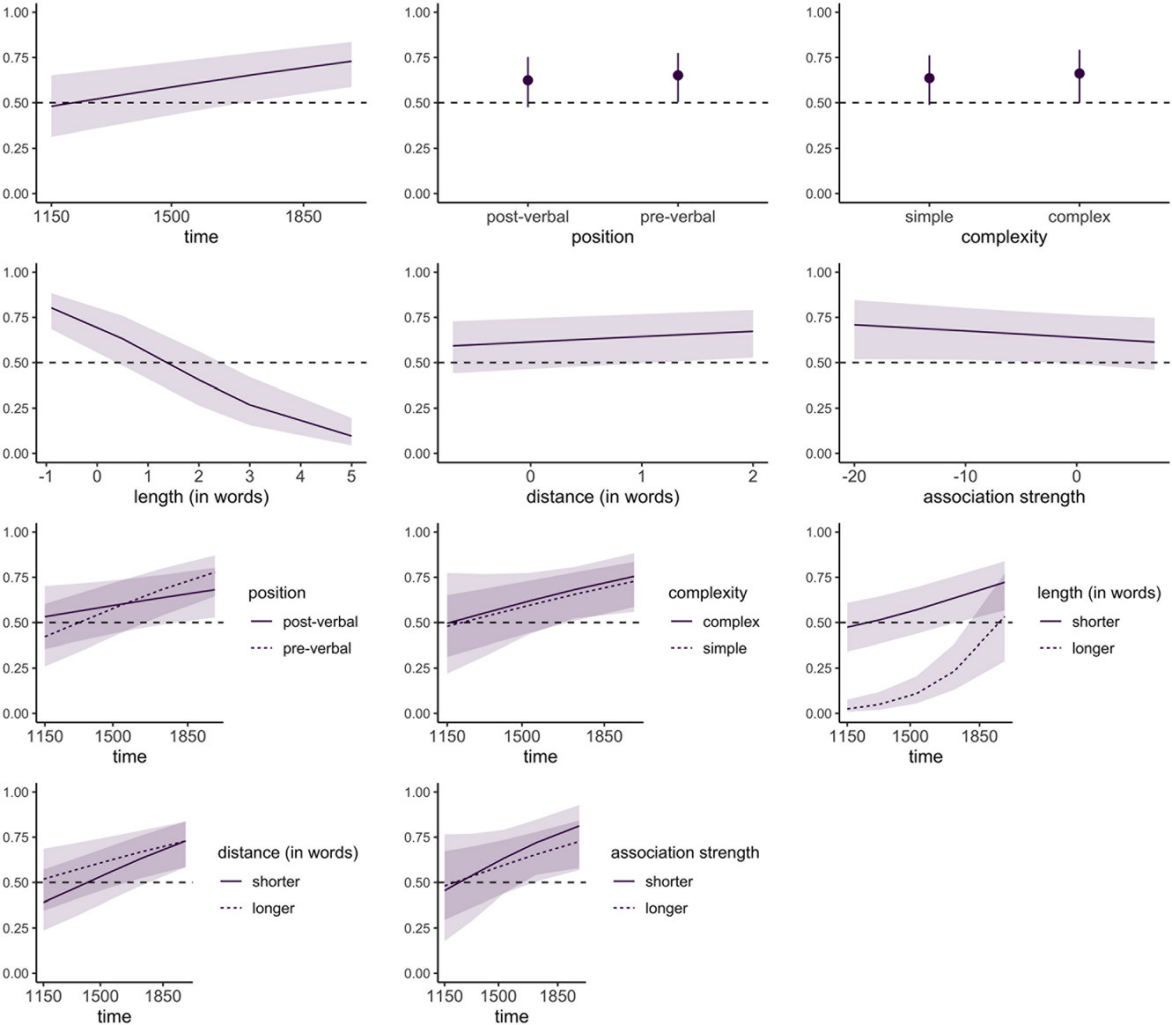
Fixed effects and interactions. The reference level is NP use, viz. higher values on the *y*-axis indicate a greater likelihood of PP expression. For interactions, the continuous variables are divided into binary categories for visualization purposes.

These results appear to be in line with the hypothesis put forward above, which is that NPs may have constituted the dominant variant in earlier English (at least in early Old English, cf. Sato 2009), but have since come to compete against and have been superseded by PPs, even though the figures may not match the great predominance of PPs observed in, for example, Quirk et al. (1985) for PDE. Nevertheless, a closer look at the distribution of variants across subperiods, decades, or individual texts suggests that this development is far from linear and straightforward, as there is great fluctuation, especially within Early Modern English. This is also in agreement with Szmrecsanyi’s (2012, 2016 point that presuming an evident change from synthetic to analytic means of expression in the history of English is too simplified a view. The next section further investigates the impact of time and its relation to other variables by means of mixed-effects logistic regression modelling as well as conditional random forests.

### 3.2 Factors impacting the alternation

In the model presented here, the dependent variable is the choice between NP or PP expression of time adjuncts; text ID as well verb lemma and head noun lemma are used as random factors, with infrequent types for the two former (<3 in the entire data set) binned into “rest” categories.1Note that such binning is common practice in linguistic regression models to avoid convergence issues, among other things (cf. e.g. Wolk et al. 2013: 399). However, as discussed in more detail in Van de Velde and Pijpops (2021: 168–169), this is not unproblematic, as infrequent factor levels are not distinguished from frequent ones, and non-independence of observations may not be represented adequately in the resulting model. Fixed effects include time and the factors outlined in (i)–(v) above: position of the NP/PP in relation to the verb, and the complexity measures of morphosyntactic complexity, length of the time expression, distance between verb and NP/PP, and association strength between verb and head noun. To assess changes in the impact of the factors over time, interaction terms between time and all fixed effects were included. Model fit is high (*C* = 0.846, Somer’s *D*_*xy*_ = 0.692), and the model is reasonably accurate (77.19 % against a baseline of 50.28 %); all variance inflation factors in the model are below 2, indicating no major issues with multicollinearity. The results of the analysis are given in Table 3 and Figure 1, showing both main effects and interactions.

**Table 3:** Mixed-effects logistic regression model output.

Random effects			
Groups	Name	Variance	Std. deviation
Text ID (552)	(Intercept)	0.848	0.921
Verb lemma (1,133)	(Intercept)	0.162	0.403
Noun lemma (14)	(Intercept)	1.245	1.116

Fixed effects						
Variables		Estimate	Std. error	*z* value	Pr(*>|z|*)	
(Intercept)		0.701	0.311	2.251	0.024	*
Time		0.093	0.069	1.347	0.178	ns
Position						
Post-verbal	Pre-verbal	0.119	0.058	2.043	0.041	*
Complexity						
Simple	Complex	0.112	0.155	0.723	0.470	ns
Length		-0.617	0.062	-9.938	<0.0001	***
Distance		0.128	0.028	4.579	<0.0001	***
Association strength		-0.016	0.013	-1.209	0.227	ns
Time: position						
Post-verbal	Pre-verbal	0.188	0.060	3.150	0.002	**
Time: complexity						
Simple	Complex	0.016	0.166	0.094	0.925	ns
Time: length		0.188	0.056	3.368	0.001	***
Time: distance		-0.065	0.028	-2.347	0.019	*
Time: association strength		-0.007	0.013	-0.576	0.565	ns

***p < 0.001. **p < 0.01. *p < 0.05.

As can be seen, time does not significantly increase the chances of the PP construction being used, meaning that the probability that PPs will be chosen over NPs is not significantly higher in later texts than in earlier texts. By contrast, regarding relative position of the time adjunct, we find that there is a significant effect on variant: PPs seem to occur more frequently in pre-verbal position, while NPs favour post-verbal position. This factor is furthermore subject to change over time, with the overall effect becoming stronger between the earliest and latest texts, and PPs becoming increasingly closely associated with initial place. These results are somewhat at odds with the tendencies observed for Present-Day English adjuncts, where both PPs and NPs exhibit similar positional biases.

Similarly, concerning the four complexity measures, the results only partly confirm expectations. First, grammatical complexity and association strength emerge as non-significant. The latter contrasts with Levshina’s (2018) study: greater predictability, meaning greater collocational strength between verb and head noun, does not seem to play a noticeable role in this case, and does not clearly increase the use of NPs over PPs. These factors also do not see any significant change in their impact over time.

Greater distance between verb and adjunct has a positive impact on PP use as anticipated, and as suggested by the Complexity Principle laid out in Rohdenburg (1996) and recently discussed with a focus on NP/PP variation in Levshina (2018). However, length of the constituent significantly decreases the probability of the PP occurring; the longer the constituent, the more often the NP is used. This finding is surprising in that greater length can be considered as reflecting greater processing complexity, and presumably also correlates with grammatical complexity (in that more complex constituents could be assumed to also be larger in size). Still, the finding may be explainable in terms of efficiency, in that the shorter NP variant may be advantageous in already long contexts (cf. also Levshina 2018). In addition, as suggested by Pijpops et al. (2018), relative position may have an important confounding impact here. Importantly, both the impact of length and of distance is subject to significant change through time – specifically, their effect appears to weaken in later texts. Again, these results are not entirely in line with the hypotheses put forward in this paper, as complexity effects were taken to be constant. They may be interpreted as indicating an expansion of PPs into all contexts regardless of complexity, but should at the same time be taken with caution, as genre differences between periods may play an important role in this regard.

**Figure 2:**
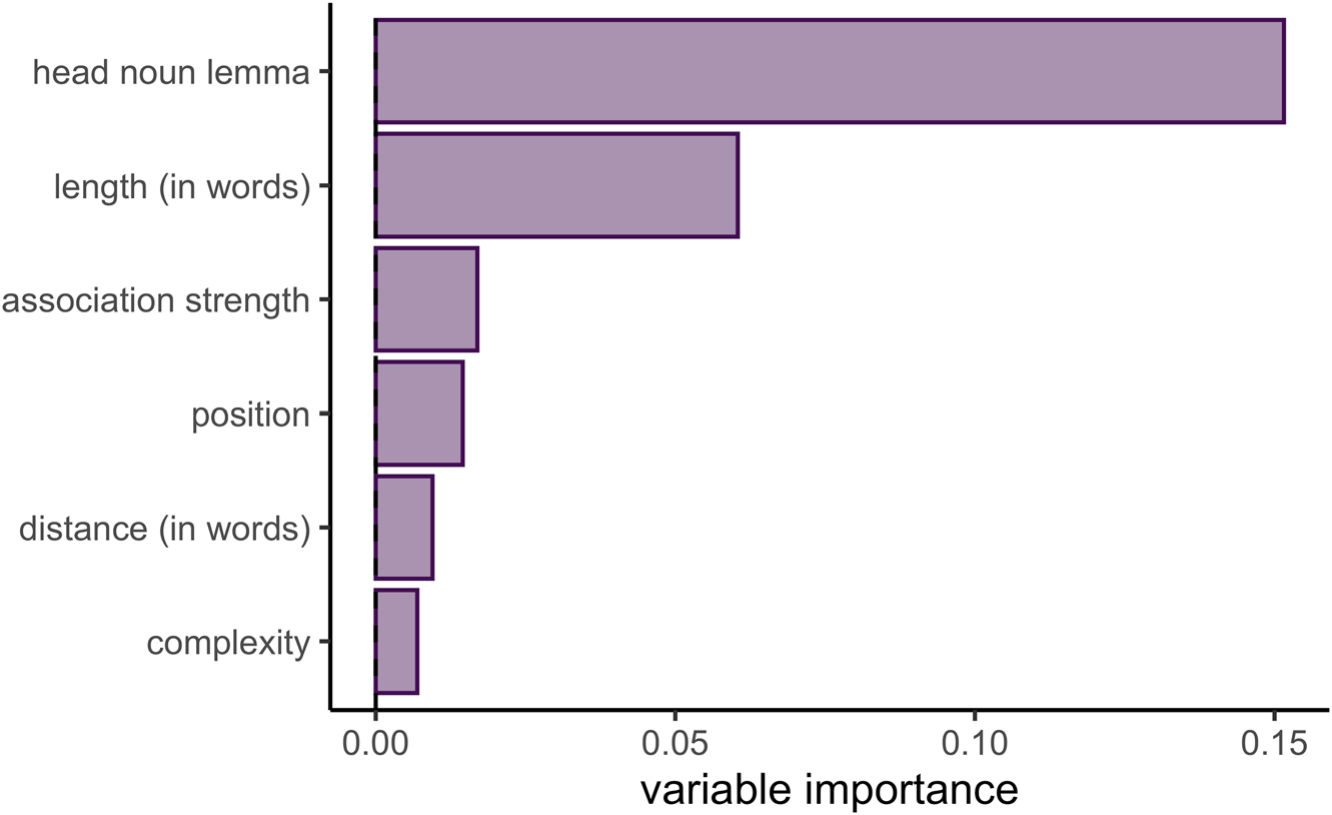
Variable importance scores for predictors (including head noun lemma in addition to complexity measures and position).

In a final step, in order to assess the impact of lexical biases in comparison to the effects of complexity and position, Figure 2 shows the results of a conditional random forest analysis, providing a ranking of variables in terms of their importance in guiding the variation between NP and PP adjuncts across the entire data set (cf. Tagliamonte and Baayen 2012; Gries 2020). As can be seen, head noun lemma greatly outweighs the other factors here – a close look at the precise frequency distributions suggest that, for example, nouns such as *afternoon*, *morrow*, *season*, *time*, and *winter* have a high likelihood of being used in PPs, whereas others like *day*, *night*, and *week* tend more towards NP use. Still others (e.g. *month*, *morning*, *noon*, *summer*, and *year*) are found in relatively equal distribution. While some nouns change in their preferences over time, separate analyses of the data for the individual periods (Middle English vs. Early Modern English vs. Late Modern English) suggest that the overall great effect of lexical information is highly stable, appearing as most influential in all models. In sum, this means that this alternation and its diachronic development is heavily dependent on the head nouns involved, and that the set of temporal nouns investigated here is not very homogeneous. To some extent, this then also calls into question whether this case of variation qualifies as an abstract alternation – this is briefly discussed in the following section, which also concludes the paper.

## 4 Discussion and conclusion

The results of the present paper raise a number of questions pertaining, on the one hand, to assumptions about the morphosyntactic history of English, and on the other hand, to the study of alternations. As to the first, the case of time adjuncts between Middle English and Late Modern English suggests that while there is some tentative support for an increase in PP use over time, the results are mixed, and the distribution fluctuates. This mirrors Szmrecsanyi’s (2012: 9) conclusion that the timespan covered here is “clearly not characterized by a steady drift toward more analyticity and less syntheticity” and that “the historical variability in English in all the historical periods, … was not particularly dramatic”. More specifically viewed in terms of diachronicity of alternation relationships, this case study accordingly does not showcase emergence or loss of an alternation, but considerable stability, or rather, stable fluctuation. This nevertheless does not preclude more subtle changes within the alternation, such as individual nouns changing in their preferences, or further evidence for a continuing “marginalization” of PPs in terms of favouring initial position, whereas NPs are more likely to appear post-verbally even when functioning as adjuncts (cf. Zehentner 2019).

Second, this case study taps into questions that are relevant to any proposed alternation. That is, while the analysis has identified influencing factors that seem to guide this alternation, it has also highlighted that lexical biases (specifically head noun preferences) are very strong predictors in this variation, and that other factors such as particular complexity measures do not impact the choice in the expected direction. This could be taken as an indication that instead of an abstract “time adjunct” alternation or even more schematic “adjunct” or “NP/PP” alternation constructions, this variation should better be considered to play out on the lower level of individual nouns (as well as potentially verbs), directly relating to Pijpops et al.’s (2021) insistence on the importance of determining the relevant level of analysis in an alternation study (see also Zehentner 2021), as well as Van de Velde and Pijpops’s (2021) discussion of lexical effects in alternations. Although not explicitly discussed in this paper, a further point to consider and incorporate in such an approach is preposition-specific preferences. Finally, this also relates to the focus on binary choices in alternation studies, which is pressing in this particular case (cf. Arppe et al. 2010). As evident in, for example, Hasselgård’s discussion of adjuncts (and also the overviews in Quirk et al. [1985] and Huddleston and Pullum [2002], among others), time references are not restricted to NPs and PPs, but can be expressed by a range of additional patterns such as adverbs or finite clauses. Reducing such multiple options to “clear-cut dichotomies” (Arppe et al. 2010: 12–13) may present a too simplistic and reductionistic perspective on this alternation and its history. Still, the present study has provided a first closer look at the development of time adjuncts and their means of expression over time, emphasizing the need for further investigation into this particular phenomenon and the larger questions at hand.
